# Superior productivity- in the laboratory and beyond

**DOI:** 10.1155/S1463924693000069

**Published:** 1993

**Authors:** Francis H. Zenie

**Affiliations:** Zymark Corporation Zymark Center Hopkinton MA 01748 USA


Journal of Automatic Chemistry, Vol. 15, No. 1 (January–February 1993), pp. 23–33

### Superior productivity – in the laboratory and beyond

**Francis H. Zenie**
*Zymark Corporation, Zymark Center, Hopkinton, MA 01748, USA*

### Gaining strategic advantage

Analytical chemistry enjoyed a revolution in instrumental techniques during the 1960s and 1970s. This, in turn, stimulated new developments of simple laboratory automation led by improved analytical instruments, autosamplers and chromatographic integrators. During the 1980s, new generations of powerful laboratory automation emerged; primarily laboratory information systems and laboratory robotics for sample automation.

In most applications, today's analytical techniques are capable of producing the required quality data. The next step is to maintain, and even raise, these standards while increasing analytical capacity. Trace analysis continues to demand greater sensitivity to measure ever-lower trace levels. Meeting this requires continuing technology improvements in sample preparation, separations techniques and analytical measurements.

### Superior productivity

Superior productivity requires earning the greatest economic value from resources; *people, time and capital.* Improving productivity is valuable today, but will become critical to survival in the future and business will continue to demand more and more from their laboratory investment. Not only must today's laboratories improve internal productivity, they must stimulate productivity throughout their company by contributing quality, timely information for critical business decisions.

Truly successful businesses require excellent technology, superior product quality and world-class customer service to achieve market leadership. This requires outstanding contributions by skilled, motivated people, thus high value-added people are essential to high value-added organizations.

Experience clearly shows:

(1) The *value* of good, timely data far exceeds the cost of determining it.
(2) The *cost* of bad data far exceeds any savings from inadequate analytical support.
(3) Problem resolution usually demands *more* data.

This paper was read at the International Symposium on Laboratory Automation and Robotics (October 1992, Boston, USA).

In *manufacturing,* for example, errors in quality data might lead to shipping unacceptable products or rejecting acceptable products. Either condition initiates an ever more costly set of potential consequences, including:

(*a*) Demotivating dedicated staff.
(*b*) Sorting good from bad.
(*c*) Rework.
(*d*) Scrap.
(*e*) Late customer deliveries.
(*f*) Customer complaints.
(*g*) Cancelled orders.
(*h*) Lost customers.
(*i*) Product recalls.
(*j*) Law suits.

If the problems continue undiscovered or unresolved, the financial and emotional costs increase exponentially; in large companies, for example, lost customers, product recalls and law suits typically cost millions of dollars.

Whilst it would be desirable to have error-free data to verify each critical step in manufacturing processes, it is too expensive to test every step thoughout the entire process. Instead, good data should be generated at key steps and introduce sufficient controls to quickly identify and solve potential problems. This leads to additional guiding principles:

(i) For effective analytical support, introduce analysis and testing prior to high value-added steps.
(ii) Turn results around quickly. Since data is generated for critical business decisions, its value decreases rapidly over time.

For example, modern adhesives are used in high value-added manufacturing such as the assembly of critical aircraft structures. It is vital to test the adhesive's characteristics prior to assembling valuable parts into a permanent structure.

In *research and product development,* analytical errors often cause large opportunity costs. That is the lost time of valuable people and the delay in reaching conclusions, either successful or unsuccessful. These opportunity costs are never recovered and they continue to grow until the error is detected, replaced with valid data and a new direction is set. In the pharmaceutical industry, for example, a successful, new ethical drug should achieve annual sales of at least $250 M. In this example, misdirected development which delays product commercialization costs the profit contribution from *$1 million revenue per day* of delay.

© Zymark Corporation 1993

### The changing role of laboratory management

As modern analytical chemistry emerged, laboratory managers needed to be technical experts in order to establish, staff and direct laboratories capable of quality analytical results. Managers spent their time training people in new technologies, acquiring modern instrumentation and developing operating procedures. Laboratories were centralized to gain access to the limited expertise in new analytical technologies and expensive instruments.

In the past, laboratory managers responded to daily demands for analytical support. Today's laboratory managers are proactive. They seek better ways to apply technology and resources to achieve business objectives.

The companies listed in figure 1, for example, have made major commitments to laboratory automation – including more powerful computers, new instrumentation and robotics and workstations for sample automation. Their experience demonstrates compelling business reasons for their investment and the significant benefits actually achieved.

Bristol-Myers Squibb, Pharmaceutical Research Institute (2)
Glaxo, Inc., Research Institute
Hershey Foods Corporation, Corporate Analytical
Johnson & Johnson, R. W. Johnson Pharmaceutical Research Institute
Miles, Inc., Consumer Healthcare Division
Pfizer, Inc., Central Research – Analytical Developement
Sandoz Pharmaceuticals, Sandoz Research Institute
Shell Development Company, Analytical Chemistry R&D
SmithKline Beecham Clinical Laboratories
Warner Lambert Company, Parke-Davis Research Division
WMI Environment Monitoring Laboratories, Inc.

*Figure 1. Laboratory automation strategic case histories.*

### Effective investing in laboratory automation

Many automation projects require significant resource investments. These investments must be justified in terms of business value and superiority over alternative investments. Typical accounting practices approach automation justification simply as a direct substitute for people performing the same task. Less quantitative benefits, such as more timely decisions, better quality, more effective people, are often excluded from the justification.

Enlightened managements recognize the limitations of traditional finanical justifications and are introducing new approaches. Judy Lewent, Vice President and Chief Financial Officer of Merck & Co., Inc., has described these new principles.

> People talk about how short-termism is driven out of the fact that financial people force discounted cash-flow ROI [Return on Investment] down the throats of everybody, and these high hurdle rates, this high cost of capital, is what stultifies investment. I just come back and say if you really understand the business, and you're a partner with the operating members of your business, then you marry the right kind of financial parameters to the business opportunities, and be an aid to the process, a strategic ally as opposed to policeman [1].

The insights and experiences of the executives who contributed their case histories, identify the objectives leading to their strategic success with laboratory automation (figure 2). The complete case histories are summarized in Appendix A. Highlighted below are brief statements illustrating the justification and benefits.

Affordable capacity for significantly higher sample loads and more complex testing per sample.

'Just-in-time analysis' for faster new product introduction, timely correction of quality problems and enhanced customer service.

Improved precision, documentation and defensible audit trails.

Significantly reduce analysis cost in order to routinely gather all the data necessary to solve problems quickly – rather than wait for more data.

Transfer valid, analytical methods to multiple sites – worldwide.

Improve motivation, reduce turnover and enhance effectiveness of valuable, scarce people.

Utilize laboratory space more effectively.

*Figure 2. Business objectives for laboratory automation*

### Affordable capacity

From 1985 through 1988, the Bristol-Myers Squibb Pharmaceutical Research Institute, increased the number of assays by 42% per year while the staffing increased by 12% per year. With 1985's productivity, it would have taken 115 more people, along with space and instruments, to perform 1988's assays. This dramatic productivity increase was accomplished with improved instrumentation, computers and laboratory robotics (see Appendix A, reference 1).

Bristol-Myers Squibb's Metabolism and Pharmacokinetics Scientific Operations was able to use the added capacity of their initial laboratory robotics system to perform about 8000 assays in-house that would have had to be subcontracted – realizing $400 000 in cost savings. Longer term, they are developing new levels of laboratory automation to double their capacity to 500 000 assays per year (see Appendix A, reference 2).

SmithKline Beecham Clinical Laboratories (SBCL) performs contract analysis as a business. SBCL projects rapidly increasing demands for testing, together with higher costs and lower prices. To meet this challenge, their competitive strategy for the year 2000 requires investment for improved employee utilization, more efficient specimen processing, improved quality and enhanced service. They invested in laboratory robotics for drugs of abuse confirmation testing after new developments demonstrated a four-fold throughput increase compared to earlier robotic approaches (see Appendix A, reference 10).

The Parke-Davis Pharmaceutical Research Division of Warner Lambert has reported a 20% annual increase in the number of stability samples and a doubling of the number of tests per sample. Laboratory automation is a key element of their stategy to meet these accelerating needs (see Appendix A, reference 11).

At Sandoz Research Institute, automated techniques increased their number of screening assays from 300 in 1987 to over 11 000 in 1992 (see Appendix A, reference 8).

Automated Dissolution Testing at Johnson & Johnson's R. W. Johnson Pharmaceutical Research Institute has saved over $1 500 000 since 1985 (see Appendix A, reference 5).

WMI Environment Monitoring Laboratories, Inc. tests ground-water samples from landfills owned and operated by Waste Management, Inc. During four years of use, their automated Chemical Oxygen Demand (COD) assays have returned approximately 600% on their investment (see Appendix A, reference 12).

### Just-in-time analysis

'Timeliness of results is our challenge for the 90s. All the return on investment justifications to purchase a robot cannot do justice to the need for timeliness . . . Timeliness in the form of robotics has replaced cost as a competitive advantage to a food company' (Hershey Foods Corporation – Appendix A, reference 4).

Miles's goal is to achieve 'just-in-time release' of products immediately following manufacture and packaging. To succeed, Miles needs more analytical data and faster sample turnaround than ever before (Miles, Inc., Consumer Healthcare Division – Appendix A, reference 6).

The Glaxo, Inc., Research Institute reported that about two years ago, management wanted to accelerate clinical trials for an important new drug. Rather than subcontract these assays, Glaxo's bioanalytical staff requested the opportunity to demonstate even faster turnaround by performing the assays in-house with laboratory robotics. Using a fully automated system, these bioanalytical assays were completed in two weeks (see Appendix A, reference 3).

'Successful robotic automation of key elements of the analytical process will lead to more timely and high quality decision-making rather than a deferral of decision-making awaiting more data . . . Improved quality of regulatory submissions will lead to faster approvals of new products. Timely in-process information will shorten the development cycle' (Warner Lambert, Appendix A, reference 11).

### Improved precision, documentation and defensible audit trails

Improved data quality and documentation are some of the most widely reported benefits of laboratory automation. A dramatic drop in cost per assay enable Squibb to provide more data with improved quality and better documentation. This investment provides Bristol-Myers Squibb with a strategic advantage in the competitive drug development business (see Appendix A, reference 1).

### Significantly reduce analysis cost

In many quality control situations, the cost of analytical data limits the amount of routine analysis. Typically, laboratories must generate additonal data in order to determine the cause of specific problems. Using two, fully automated content uniformity systems, Miles staff routinely generates sufficient data, in virtual real time, to correct problems quickly and ensure quality production (see Appendix A, reference 6).

### Transfer valid analytical methods to multiple sites

Hershey uses laboratory robotic automation in their central analytical laboratories and has transferred the technology to the main plant laboratories and in-plant mini-labs (see Appendix A, reference 4).

These results triggered a new confidence in laboratory robotics technology. Today, it is the approach of choice. US laboratories are training Glaxo people, from around the world, on the effective use of the technology (see Appendix A, reference 3).

### Improve motivation, reduce turnover and enhance effectiveness of people

'When a robot is used, one can reasonably expect to see dramatic improvements in safety, productivity and precision. Even more important is the improvement in quality of life for employees, since robots do best those kinds of jobs which are seldom interesting or challenging for humans' (Shell Development Company – Appendix A, reference 9).

'This dramatic success generated even greater commitment and additional strategic benefits, including . . . energizing existing staff and attracting new people as work became less routine and more challenging' (Glaxo, Inc. – Appendix A, reference 9).

'The purpose of robotics is to gain throughput, relieve staff of repetitive tasks, permit them to use their greatest tool, their brain, and most important of all, to yield routinely precise and accurate results in a timely manner' (Hershey Foods Corporation – Appendix A, reference 4).

Adopting automated drug-screening techniques at Sandoz Research Institute, dramatically enhanced the job content for bench scientists (see table 1):

*Table 1. Drug screening at Sandoz Research Institute.*

| *Bench scientist activity* | *1987* | *1991* |
|---|---|---|
| Analysis and data management | 54·0% | 10·0% |
| New assay development and personal skill development | 46·0% | 90·0% |

See Appendix A, reference 8.

Determine the real business for automating this method or methods.

Quantify the economic value of these benefits.

Short-term labour savings are typically a reduction of investment – rather than the primary benefit.

If significantly lower 'cost of analysis is the primary benefit, calculate savings on the projected sample loads. Compare savings to actual experience not ideal models.

If this is new technology for you, consider a 'facilitating investment' by amortizing the learning and start-up costs over potential projects.

*Figure 3. Systematic approach to justifying laboratory automation.*

### Utilize laboratory space more efficiently

Today, virtually all laboratories are space limited and valuable laboratory space is now a strategic resource. The reasons for this include:

(1) It is expensive and must be added in large increments – people can be added incrementally, but space must be added in economical units. Even with sufficient land, the smallest practical new laboratory construction would be 30 000 to 50 000 square feet. This requires an investment of between $6 and $10 M. The relocation costs need to be added.
(2) Long lead time: a decision to construct new laboratory space must be made 18 to 24 months, or more, before it is needed.
(3) Requires extensive management time: successful laboratory expansions require management time to ensure that plans match strategic needs, plan layouts, redeploy people and instrumentation and negotiate conflict generated by the expansion.

If the average cost of laboratory space is $150 to $200 per square foot, the 'real' cost of usable bench space will be in the range of $750 to $1500 per linear foot.

### Justifying laboratory automation: calculating direct cost savings

The decision to invest in laboratory automation should be based meeting the stategy and financial needs of the business. Today's executives must place a value on intangibles and make them part of a business justification. Begin justification with the guidelines highlighted in figure 3.

Whether saving direct people cost is the primary justification or a side benefit, it's important to make valid calculations of the projected savings.

The first step is to determine average hours saved per unit of work. To do this, determine the actual effort currently expended, not the theoretical or ideal goal. Next, project the effort required to perform the work using the proposed automated method. The difference becomes the man-hours saved per unit of work. Finally, convert this to savings per week using the amount of work performed, or projected, each week.

*Calculating direct cost savings: example*

*Step 1. Calculating real cost. Example: annual cost of senior technician*

| Direct cost elements | Annual cost |
|---|---|
| Annual salary – no overtime | $30 000 |
| Direct benefits | 10 000 |
| Medical, life, FICA, disability, workers compensation, unemployment, retirement, 401K, Profit sharing | |
| Space & utilities – 200 sq. ft. | 2000 |
| Communication, travel, training | 1500 |
| Other | 1500 |
| Total direct cost | $45 000 |

Note: Senior staff may have higher salaries and benefits are more costly in many companies. Costs do not include, supervision, administrative support overhead or profit.

*Step 2. Analysis of days available to perform job assignment*

| Category | Days |
|---|---|
| Total working days per year | 260 |
| Less, equivalent days not available | |
| Vacation | 13 |
| Sick | 5 |
| Holidays | 10 |
| Training | 5 |
| Travel | 3 |
| Breaks & distractions at 1 hour per day | 30 |
| Administrative – meetings, supervision, reports | 14 |
| Total not available | 80 |
| Days not available to perform job assignment | 180 |

Note: This is 70% of total time, which considered excellent.

*Step 3. Summary*

Available hours to perform the job assignment is 180 days x 8 hours per day = 1440 hours.
The cost per hour actually performing the job assignment is $45 000 annual cost divided by 1440 hours available per year = $31.00 per hour.
The annual savings from saving 1 hour per day performing the job assignment is 250 hours per year at $31.00 per hour = $7500.
Therefore, by saving 1 hour per day:

A $7500 investment will be recovered in 1 year.
A $15 000 investment will be recovered in 2 years.

In many organizations, the annual cost per person is much higher than this example and fewer days are available to perform the job assignment. Under these conditions, paybacks can be far faster than shown above.

### Conclusion

The strategic challenges caused by world-wide competition and regulation continue to increase. People and time are our most precious resources.

Modern laboratories must become a catalyst and role model stimulating greater productivity throughout their organizations. Laboratory managers must grow as business executives bridging the laboratory to strategy. Laboratory automation technology has demonstrated its ability to significantly enhance productivity in the laboratory and beyond.

### Reference

1. *Wall Street Journal* (30 June 1992), p A17.

### Appendix A

*Reference 1 (October 1991)*

*Bristol-Myers Squibb: Glenn A. Brewer, Executive Director, Worldwide Analytical Systems, Bristol-Myers Squibb, Pharmaceutical Research Institute, New Brunswick, NJ, USA*

In 1984, Squibb (now Bristol-Myers Squibb) Research Institute anticipated rapidly increasing needs for analytic assays to support their drug development programme. Increasing demand for content uniformity, dosage form stability and body fluid assays was stimulated by multiple forces; including, accelerated research and development, internal research demands for more data and changing regulatory requirements.

Squibb managers and scientists determined that only a strategic commitment to sample preparation automation, advanced analytical techniques and powerful data management would meet their needs.

They convinced senior management, organized for automation, established a critical mass of talent and equipment and developed methods to take full advantage of the new technology. This total commitment delivered impressive results.

| | *1985* | *1986* | *1987* | *1988* |
|---|---|---|---|---|
| Laboratory staff | 79 | 87 | 105 | 110 |
| Total routine assays | 35 177 | 51 702 | 85 476 | 101 256 |
| Assays per person | 445 | 594 | 814 | 921 |

During this period, staffing increased 12% per year while total routine assays increased 42% per year. In order to perform 1988's assays at 1985's productivity, Squibb would have had to add 115 more people along with sufficient laboratory space and instrumentaion.

The dramatic drop in cost per assay enabled Squibb to provide more data with improved quality and better documentation. This investment provides Bristol-Myers Squibb with a strategic advantage in the competitive drug development business.

Bristol-Myers Squibb Case History 1.

*Reference 2 (October 1992)*

*Bristol-Myers Squibb: Raymond H. Farmen, Director, Metabolism and Pharmacokinetics Scientific Operations, Bristol-Myers Squibb Pharmaceutical Research Institute, Syracuse, NY, USA*

Bristol-Myers Squibb's Pharmaceutical Research Institute has extensive laboratory operations in Syracuse, New York and New Brunswick, New Jersey. Their Metabolism and Pharmacokinetics (MAP) Scientific Operations are responsible for bioanalytical assays radiochemical synthesis and computer data systems in support of pre-clinical and clinical studies.

Several years ago, MAP Scientific Operations anticipated dramatic increases in the number of bioanalytical studies required for new drug approval. To meet this challenge, they pioneered the use of new instrumentation, computers, laboratory robotics and automation workstations for bioanaltyical assays. By most standards they are highly automated today, and yet, they are planning even more powerful laboratory automation capability to meet the expected work load three to five years in the future. Today MAP Scientific Operations perform about 300 000 assays per year in their laboratories and in contract laboratories. Their goal is to develop capacity to perform 500 000 assays annually.

The following example highlights the effective use of both robotic systems and automation workstations at their Syracuse, New York laboratories to support Bristol-Myers Squibb's NDA for a new antidepressant, nefazodone. They developed a liquid/liquid extraction procedure for three metabolites. Later, a second liquid/liquid extraction method was developed for a fourth metabolite, thus requiring two liquid/liquid assays per sample. These liquid/liquid extractions were performed by a Zymate robotic system. To reduce the number of assays, they developed a manual solid phase extraction (SPE) method capable of measuring all metabolites in a single assay. This SPE method was transferred to a BenchMate Workstation to handle the growing sample load. Since then, the laboratory has added a second BenchMate Workstation and a second Zymate robotic system capable of both liquid/liquid and SPE extractions.

The added capacity provided by laboratory automation permitted internal running of the increased sample load – by existing staff. The initial robotic system generated these cost containment savings during 1990 and 1991.

Looking forward, MAP Scientific Operations plan to further increase capacity for even more bioanalytical assays; generated by expanded new product development regulatory requirements for more data and the routine assay of more standards made possible by automation. Their goal is to gain a 10-fold increase in assays per analyst by investing in laboratory instruments, computers and automation – supported by a highly skilled staff of automation scientists. In addition, they expect to decrease the typical study turn-around time from six to eight weeks down to one to two weeks. By substantially reducing assay cost and study turn-around time, this centralized laboratory will attract bioanalytical work from Bristol-Myers Squibb's laboratories around the world.

MAP Scientific Operations recognize the technology and management challenges ahead. They are also energized by the exciting career development opportunities being created.

Cost containment from robotic automation

Samples analysed during 1990 and 1991 with robotics: 18 306 samples

Estimated sample capacity using manual methods: 10 800 samples

Samples assayed in-house that would have required contract laboratory service: 8266 samples

Typical contract laboratory fee: $50 per sample

Contract laboratory savings (1990 and 1991): (8226 samples) ($50 per sample) = $411 300

Bristol-Myers Squibb Case History 2.

*Reference 3 (October 1991)*

*Glaxo, Inc. Research Institute: Julie J. Tomlinson, Robotics Laboratory Manager, Clinical Pharmaceutics, Research Triangle Park, NC, USA*

Glaxo, a leading multinational pharmaceutical company, has major research and development laboratories in the UK and the USA, with smaller laboratories around the world. Rapid new product development is a vital element of Galxo's business strategy. Bioanalytical assays for drug metabolism studies are a critical factor in Glaxo's development process.

Glaxo was an early user of laboratory robotics for drug metabolism with limited success, and senior management was cautious about full commitment to this new technology. Up to 70% of their bioanalytical assays were performed by contract laboratories.

About two years ago, management wanted to accelerate the clinical trials for an important new drug. Rather than subcontract these assays, Glaxo's bioanalytical assay staff requested the opportunity to demonstrate even faster turnaround by performing the assays in-house with laboratory robotics. Using a fully automated system, these bioanalytical assays were completed in two weeks.

These results triggered a new confidence in laboratory technology. Today, it is the approach of choice. Seven automated methods are performed routinely on four robotic systems. Only 30% of their bioanalytical assays are now subcontracted. And, the US laboratories are training Glaxo people, from around the world, on the effective use of this technology.

This dramatic success generated even greater commitment and additional strategic benefits, including:

(1) Effective methods transfer throughout Glaxo's worldwide laboratories.
(2) Energizing existing staff and attracting new people as work became less routine and more challenging.
(3) Improved analytical precision particularly through use of gravimetric techniques.
(4) Lower cost of analysis to meet the analytical demands of increasing regulation.
(5) Greater safety for people working with human biological samples.

*Reference 4 (October 1991)*

*Hershey Foods Corporation: Robert A. Martin, Jr., Sr. Manager, Corporate Analytical Laboratories, Hershey, PA, USA*

Hershey Foods Corporation is a leading food company with one of the most advanced analytical capabilities in the food industry. Increasing demands for quality, labelling and safety assessment make today's food laboratory a strategic resource.

Robert Martin presented the following ideas at the Food Labs '91 Conference:

> 'Hershey uses laboratory robotic automation on their central analytical laboratories and have transferred the technology to the main plant laboratories and in-plant mini-labs. Their automation goal is not replacing people. Rather . . . The purpose of robotics is to gain throughput, relieve staff of repetitive tasks, permit them to use their greatest tool, their brain, and most important of all, to yield routinely precise and accurate results in a timely manner.
>
> A major challenge to the food laboratory in the 1990s will be to incorporate the equivalent of just-in-time delivery of inventory and manufacture of finished goods. This is just-in-time results . . . This is where robotics earns its keep; it frees staff to persue new technologies and react to one-off emergency situations to produce timely results.
>
> Timeliness of results is our challenge for the 90s. All the return on investment justifications to purchase a robot cannot do justice to the need for timeliness . . . TIMELINESS in the form of robotics has replaced COST as a competitive advantage to a food company'.

In a recent communication, Dr Martin summarized his thoughts: 'Laboratory robotics is not just a good idea, it is a necessary reality to address the demands placed on the laboratory of the 1990s'.

*Reference 5 (October 1992)*

*Johnson & Johnson: Irwin Gibbs, Director, Pharmaceutical Development, R. W. Johnson Pharmaceutical Research Institute, Spring House, PA, USA*

The Pharmaceutical Development department at Johnson & Johnson's R. W. Johnson Research Institute provides solid dosage form dissolution testing services for many of J&J's pharmaceutical development projects. They support formulation development, clinical study supplies, process development and process scaleup. The number of dissolution sample batches remained relatively constant during the 1980s, but began increasing in 1990. The pharmaceutical development staff anticipated the increasing work demands and, in 1985, installed their initial laboratory robotics system to automate dissolution testing.

As they gained experience, a growing percentage of their dissolution testing was converted from manual to automated processing. By 1991, 90% of all dissolution testing was performed by three automated systems. During 1991, this saved 52% in man-days leading to financial savings over $500 000. From 1985 through 1991, the cumulative savings earned by automated dissolution testing exceeded $1 500 000.

Their three systems are virtually identical and are capable of running a wide range of dissolution assays. This automation not only increases assay capacity, but also automatically generates easily retrieved documentation for future reference.

J&J's approach to automated dissolution testing encouraged the rapid development and validation of automated methods and companion manual methods. The companion methods ensure a validated fall back alternative and also provide regulatory agencies with a valid method which can be performed by a non-automated laboratory.

Irwin Gibbs, Director of Pharmaceutical Development, states; 'The success of laboratory robotics in automating dissolution testing has lead the way towards future efforts to automate other laboratory testing in Pharmaceutical Development at the R. W. Johnson Pharmaceutical Research Institute.

*Reference 6 (October 1991)*

*Miles Inc., Consumer Healthcare Division: Bernd C. Schade, Director of Quality Assurance, Elkhart, IN, USA*

Miles Consumer Healthcare Division, a large manufacturer of over-the-counter pharmaceutical products, has installed fully automated content uniformity testing for

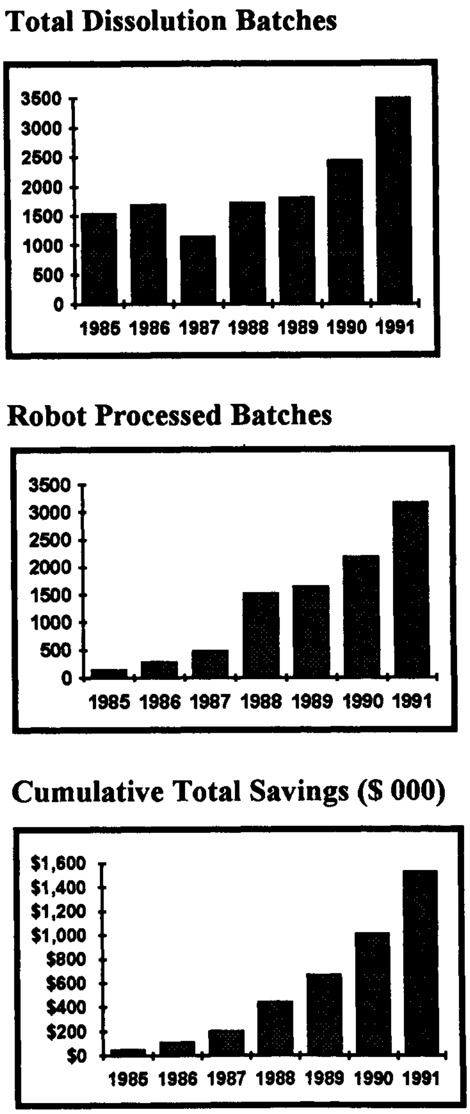

*Figure 4.*

its Alka-Seltzer family of antacid and cold-relief tablets. Miles produces over 10 M Alka-Seltzer tablets each day using seven formulations.

Miles' goal is to achieve 'just-in-time release' of products immediately following manufacture and packaging. To succeed, Miles needs more analytical data and faster sample turnaround than ever before. In essence, Miles is approaching traditional, final product quality-control at a process control challenge.

In many quality control situations, the cost of analytical data limits the amount of routine analysis. Typically, laboratories must generate additonal data in order to determine the cause of specific problems. Further in high volume production, the time delay in obtaining sufficient data leads to continuing production of large quantities of potentially unsatisfactory product.

Today, Miles has two fully automated content uniformity systems for Alka-Seltzer quality assurance. The dual capability ensures adequate testing capacity, rapid sample turnaround and back-up reliability. Miles's staff routinely generates sufficient data, in virtual real time, to correct problems quickly and ensure quality production. Their automation investment significantly reduced cost per analysis and, beyond this, they gained greater production productivity through lower scrap, reduced sorting of finished products and more rapid inventory turnover.

Alka Seltzer is a registered trademark of Miles Inc.

*Reference 7 (October 1992)*

*Pfizer, Inc.: Central Research Division, James E. Curley, Assistant Director, Analytical Research & Development, Groton, CT, USA*

Pfizer scientists in Analytical Research & Development pioneered in the use of laboratory robotics beginning in 1983. They have applied robotic automation to tablet dissolution testing, tablet assays and dosed feed analysis. Senior management recognized the growing need for analytical support and the importance of relieving skilled scientists from routine work. They identified laboratory robotics as a new technology with the potential to meet these needs.

Tablet dissolution testing was selected for automation because regulatory trends require dissolution rate specifications for tablets and capsules. Early testing was based upon the premise cited in the USP that a single-time point determination at 45 minutes would assure therapeutic acceptability if the product was 75% dissolved. As the technology has become more extensively used, dissolution rate testing has taken on an additional role of assuring that manufacturing processes remain in control. Today, dissolution rate testing is included in a series of tests that 'fingerprint' the quality of a dosage form. During drug development, comprehensive dissolution studies are preformed for multiple dosage formulations to profile dissolution rate and stability. For immediate release tablets, routine dissolution assays continue to be single point, but sample timing is selected to provide the most information about that specific drug formulation.

The more demanding uses for dissolution data, require even greater control over assay parameters such as media composition, temperature and dissolved gas. This, in turn, requries more powerful automation to measure and control these parameters as well as perform the actual test.

Pfizer has standardized their automated dissoluton approach such that their robotic systems become virtual workstations. Early attempts to have sample submitters directly interface with the systems were unsuccessful so Analytical Research provides skilled operators to set up and run samples.

Dosed feed assays for safety assessment and metabolism studies, are also successful laboratory robotics applications. Early in the development of a drug candidate, there may not be sufficient time to develop an automated analytical method to support safety studies. The goal, however, is to use a robotic system by the time the drug candidate is in long-term safety studies.

Tablet assay automation, however, has not met Pfizer's expectations. They desire a standardized, robust and compact automated workstation. Efficient laboratory space utilization has become a strategic factor in Pfizer's laboratory automation programmes.

*Reference 8 (October 1992)*

*Sandoz Pharmaceuticals, Sandoz Research Institute: David B. Weinstein, Director Dennis S. France, Senior Scientist, Atheroscleroris and Vascular Biology Research, East Hanover, NJ, USA*

Sandoz Pharmaceuticals is a leading, multi-national developer and producer of pharmaceutical and healthcare producs. In his keynote presentation at the 1991 International Symposium on Laboratory Automation and Robotics, David Weinstein described how robotics accelerated their drug discovery research and stimulated a more creative scientific environment.

Dr Weinstein described a new environment in which laboratory robotics plays a key role: 'The role of the research director who is the product champion is to blend together the research personnel components with the creative environment provided by mangement in order to create the successful and stable integration of robotics into the early stages of drug discovery . . . At Sandoz Research Institute, we have created an opportunity for enterprising biologists to develop a drug discovery programme that is heavily based on robotic microplate management systems as analysis tools. This programme was accomplished with minimum amounts of funding and a staff of no more than two over a two-year period'.

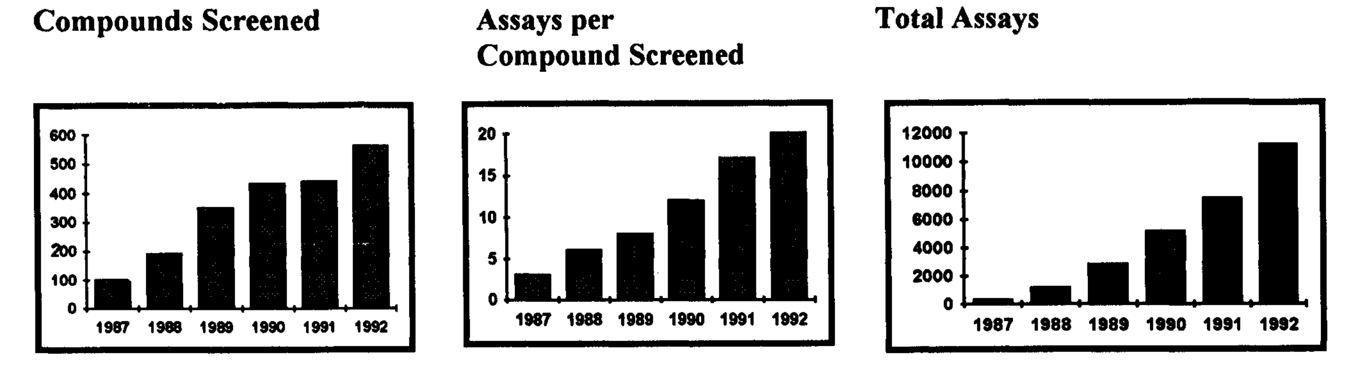

*Figure 5.*

There are typically three stages in the rational drug discovery process:

(1) Determine the biological site or mechanism of action to target.
(2) Develop *in vitro* and *in vivo* test models, which can discriminate between compounds with effectively alter the selected biological target.
(3) Assign large numbers of medical chemists to provide rational concepts toward design of complicated chemical molecules which may achieve the desired biological response.

Sandoz have realized a dramatic increase in screening assays from their manual assay capability in 1987 through installation of the first robotic system in 1988 and a second system in 1990. These assays are from complex *in vivo* screens which increased from 100 compounds tested in 1988 to well over 500 in 1992. More importantly, automation enabled them to increase the number of assays per compound from three in 1988 to 20 in 1992. The total number of assays, therefore, increased 35 fold from 300 in 1987 to over 11 000 in 1992.

At Sandoz, laboratory robotics provides a powerful tool to automate laboratory screening and, in doing so, frees up valuable time of skilled scientists. This is illustrated by the change in bench scientist activity at Sandoz between 1987 and 1991.

| *Bench scientist activity* | *1987* | *1991* |
|---|---|---|
| Analysis and data managment | 54·0% | 10·0% |
| New assay development | 32·0% | 40·0% |
| Staff meetings | 10·0% | 20·0% |
| Scientific meetings | 4·0% | 15·0% |
| Management functions, training and job enrichment | 0·0% | 15·0% |

While significantly more bioanalytical work is performed, the scientific staff is able to perform high value-added functions, develop new scientific knowledge and participate in managment functions.

Dr Weinstein has explained that this rational approach is often balanced by the traditional pharmaceutical programme of 'random-screening' of natural sources such as bacterial and fungal broths, plant extracts and natural herbal remedies. To be most productive, random-screening requires effective screening mechanisms followed by laboratory screening of large numbers of candidate samples.

Large pharmaceutical companies typically have compound libraries containing tens of thousands of pure compounds, most of which are in sufficient quantity routine for screening. In addition to the complex *in vivo* screens, therefore, Sandoz also performs random, *in vitro* assays for enzyme and receptor targets. Typically, in random screening programmes, the rate limiting steps are compound weighing dissolving.

Recently, the Atherosclerosis and Vascular Biology department applied laboratory automation to give Sandoz scientists improved access to the Sandoz Research Institute compound library. Using a BenchMate robotic workstation they:

(*a*) Automatically tared 200 test tubes.
(*b*) Manually transferred a non-quantitative amount of compound.
(*c*) Automatically weighed tubes and calculated net compound weight.
(*d*) Automatically dispensed the appropriate amount of water and DMSO to yield a final concentration of 1·0 mg/ml and 50% DMSO.
(*e*) Automatically downloaded compound weights to an Oracle database which updated the compound inventory.

Based on weighing and dissolving 6000 compounds, they averaged 60 compounds per person-hour – a 10-fold improvement over prior manual methods.

Future automation plans at Sandoz include automating radioactive assays and high speed, automated pipetting to make replicate samples from the compound library.

*Reference 9 (October 1991)*

*Shell Development Company: Glenn L. Taylor, Director, Analytical Chemistry R&D, Houston TX, USA*

In his keynote presentation at the 1990 International Symposium on Laboratory Automation and Robotics. Glenn Taylor highlighted the strategic role of laboratory robotics at Shell Development Company: 'Analytical chemistry is being revolutionized by new capabilities conferred by the computer. At the same time, business has to face a new set of environmental and regulatory demands plus new competing pressures. Concurrent with this is a culture stressing quality and continual improvement, and in-depth fulfillment of customer requirements. In analytical chemistry, this translates to a need for more accurate, more rapid and more cost-effective analytical service'.

There are many forms of successful laboratory automation: 'Robots are probably the most complex of these approaches, and should be used only when the other forms of automation will not suffice. However, when a robot is used, one can reasonably expect to see dramatic improvements in safety, productivity and precision. Even more important is the improvement in quality of life for employees, since robots do best those kinds of jobs which are seldom interesting or challenging for humans'.

Specifically, Shell reports:

(1) The first robot at Shell has handled over 40 000 samples since 1984. It paid for itself within four months and requires no more than about one hour of setup and maintenance per day.
(2) Payback periods have been six months or less for several applications.
(3) Some environmental applications have been successfully duplicated at operating locations.
(4) Robotic applications have helped minimize worker exposure to hazardous chemicals and reagents.

Shell expects the number of quantitative analyses in which robots are involved, one way or another, to increase from approximately 20% of the analyses in 1990 to over 50% by 1995. In conclusion, our experience at Shell with robotics has been a very positive one. In every case, we can identify benefits that were not foreseen in the original justification of the robotic equipment.

*Reference 10 (October 1991)*

*SmithKline Beecham Clinical Laboratories: David O'Bryan, Vice President, Production Support Services, King of Prussia, PA, USA*

SmithKline Beecham Clinical Laboratories (SBCL) is the leading independent clinical testing laboratory in the United States. Analysing samples is their business.

The clinical testing industry is facing strategic opportunities and challenges.

(1) Clinical testing will increase due to our aging population.
(2) Government and third party reimbursements are decreasing.
(3) Large, efficient laboratories are likely to be the primary provider of sophisticated tests in the future.
(4) The supply of trained laboratory staff is rapidly falling behind industry needs while salaries and benefits are increasing.
(5) Regulatory requirements continue to increase.

SBCL believes that larger central laboratories can be more cost effective and afford the technology skills and automation investment necessary to meet these challenges. Their competitive strategy for the year 2000, includes:

(*a*) Improve employee utilization.
(*b*) Participate and assist in laboratory consolidation.
(*c*) Invest in capital technology to improve quality and enhance service.
(*d*) Improve the entire testing process from specimen acquisition and ordering to results entry and filing.

A growing business segment for SBCL is drugs of abuse testing. Following initial screening, positive screens are confirmed by GC/MS. GC/MS confirmation testing requires extensive sample pre-treatment prior to analysis; and commercially available clinical analysers are unsuited to these pre-treatment procedures. To satisfy their needs, SBCL determined that automated sample prep was necessary and that it must meet NIDA regulations, deliver near term savings and become a platform for long term competitive advantage. While laboratory robotics had demonstrated capability to perform quality sample prep for drugs of abuse confirmation, existing capabilities fell short of SBCL's throughput requirements. Detailed discussions between SBCL and Zymark led to a new approach. Utilizing Zymark's new XP Robot, advanced control techniques and independently functioning peripherals, it met all requirements. Zymark and SBCL collaborated on this development and, today, two systems are installed in SBCL laboratories.

*Reference 11 (October 1991)*

*Warner Lambert Company: Mahdi B. Fawzi, Vice President of Product Development, Parke-Davis Pharmaceutical Research Division Morris Plains, NJ, USA*

In his keynote presentation at the 1990 International Symposium on Laboratory Automation and Robotics, Dr Fawzi described how laboratory automation is a strategic resource for meeting the regulatory and competitive demands of the pharmaceutical industry: 'The complexity of new drugs and delivery systems will increase, and the characterization required for pharmaceutical products of the future will be far greater than for conventional drugs and delivery systems. Recruitment and retention of pharmaceutical product development scientists requires enrichment of the job. In our industry, the first company to gain approval and market new products, usually captures the largest market share'.

At Warner Lambert:

(1) The number of samples requiring analysis increased 20% per year from 1988 to 1990.
(2) The number of tests per stability sample more than doubled between 1985 and 1990.
(3) The cost for domestic stability testing of samples requiring HPLC analysis range from $400 000 to $600 000.

'Successful robotic automation of key elements of the analytical process will lead to more timely and higher quality decision-making rather than deferral of decision-making awaiting more data. Improved quality of information and prompt turnaround are expected to improve decision making . . . Improved quality of regulatory submissions will lead to faster approvals of new products. Timely in-process information will shorten the development cycle. Robotic automation has the necessary flexiblity to turn this vision into reality'.

*Reference 12 (October 1992)*

*WMI Environmental Monitoring Laboratories, Inc.: Deborah C. Hockman, President, Geneva, IL, USA*

WMI Environmental Monitoring Laboratories, Inc. (WMI-EML) is a wholly owned company of Waste Management, Inc. a worldwide leader in environmental services. WMI-EML tests ground-waste samples utilizing state-of-the-art facilities and equipment, including robotic automation, to meet EPA requirements. The samples are obtained from landfills and other facilities owned and/or operated by Waste Management.

While many analytical laboratories operate as corporate cost centres, WMI-EML operates as a profit centre and must be competitive with independent testing laboratories in terms of price, service and quality. Waste Management created WMI-EML as a centralized groundwater testing laboratory supporting their landfills throughout the US and Canada. The large number of samples (nearly 200 000 in 1991), in similar matrices, made the laboratories ideal candidates for robotic laboratory automation.

As a business, WMI-EML evaluates capital investments by calculating the payback period required to recover the investment. For automation projects, the fixed cost includes system cost, set-up cost and applicable overhead. The variable cost per sample includes operator cost, overhead, chemicals and miscellaneous. The break-even point is then the number of samples where the total fixed plus variable costs equal the revenue.

The initial laboratory robot installed at WMI-EML was applied to Chemical Oxygen Demand (COD) assays. COD assays were selected as the first robotic application because of the rapid payback potential. For that application, the break-even point was determined to be about 4000 samples which represented about eight months work. During its four years of use, the total return on investment is approximately 600%.

The accuracy of the robotic system compared to manual methods, shown opposite, was evaluated in a blind study using samples from the Wisconsin Department of Natural Resources. Data in mg/l.

| | *Robotic method* | *Manual method* |
|---|---|---|
| | 112 | 94 |
| | 105 | 91 |
| | 109 | 94 |
| | — | — |
| Average | 109 | 93 |
| Recovery | 98% | 84% |

The three robotic systems currently in use at WMI-EML have delivered:

(1) Rapid financial payback
(2) Equal or better data quality compared to manual procedures.
(3) Legally defensible documentation.
(4) Rapid sample turn-around-time. For example, the COD system can process 120 tests, operating unattended, during a 24-hour period.
(5) Improved staff utilization, including the transfer of human effort to innovation instead of mundane tasks.

Dr Hockman concluded that: 'Laboratory robotics is a cost effective, automation tool . . . as essential to the modern laboratory as a LMS or autosampler on an instrument'.

